# The complete mitochondrial genome of *Argiope ocula* (Araneae: Araneidae) and its phylogeny

**DOI:** 10.1080/23802359.2019.1673251

**Published:** 2019-10-03

**Authors:** Yi Yan, Kang-Kang Xu, Da-Xing Yang, Can Li, Wen-Jia Yang

**Affiliations:** Guizhou Provincial Key Laboratory for Rare Animal and Economic Insect of the Mountainous Region, College of Biology and Environmental Engineering, Guiyang University, Guiyang, China

**Keywords:** *Argiope ocula*, Araneidae, mitogenome

## Abstract

The complete mitochondrial genome of *Argiope ocula* (GenBank accession number MN331657) is a circular molecule of 14,079 bp in length, comprising of 13 protein-coding genes (PCGs), 22 transfer RNA genes (tRNAs), two ribosomal RNA genes, and a control region. Ten intergenic spacer regions and 19 reading frame overlaps were found in the mitogenome of *A. ocula*. Ten tRNAs (*trnD*, *trnF*, *trnK*, *trnR*, *trnT*, *trnW*, *trnE*, *trnG*, *trnL_1_*, and *trnM*) lacked the TΨC arm stem, whereas three tRNAs (*trnA*, *trnS_1_*, and *trnS_2_*) lost the dihydrouracil (DHU) arm. Phylogenetic analysis suggested that *A. ocula* had the closest evolutionary relationship with *A. perforata*.

The orb-weaver spider, *Argiope ocula* (Araneae: Araneidae), is a major predator of many economical agricultural pests and distributed widely in farmland and orchard (Levi 2004; Platnick 2015). In this study, adult species of *A. ocula* were collected from Maolan Nature Reserve in Libo county, Guizhou Province, China (N25°18′, E107°52′), and deposited in the spider specimen room of Guiyang University with an accession number GYU-GZML-01. The complete mitochondrial genome of *A. ocula* (GenBank accession number MN331657) is a circular molecule of 14,079 bp in length, comprising of 13 protein-coding genes (PCGs), 22 transfer RNA genes (tRNAs), two ribosomal RNA genes (*rrnL* and *rrnS*), and a putative control region. All genes have the similar location and strands with that of other determined spiders (Li et al. 2016; Yang et al. 2019). The overall base composition of *A. ocula* mitogenome was biased towards A and T, with 75.07% of A + T content (A = 36.35%, T = 38.72%, C = 9.57%, G = 15.36%). The AT-skew and GC-skew of this genome were −0.032 and 0.232, respectively.

Fifteen genes was transcribed on the minority strand, whereas the others were oriented on the majority strand. Gene overlaps in the *A. ocula* mitogenome were found at 19 gene junctions and involved a total of 180 bp, the longest 13 bp overlapping located between *trnE* and *trnF*. There are 10 intergenic spacer regions in a total of 55 bp and the largest spacer (8 bp) resided between *trnA* and *trnN*. Eight PCGs start with the typical ATN codons (ATT for *cob*, *nad2*, *nad5*, and *atp8*; ATA for *atp6*, *nad3*, *nad4*, and *nad4L*), three PCGs (*cox2*, *cox3*, and *nad6*) start with TTG, and two PCGs (*cox1* and *nad1*) use TTA as initiation codon. Eleven PCGs (*atp6*, *atp8*, *cob*, *cox1*, *cox2*, *cox3*, *nad1*, *nad2*, *nad4*, *nad4L*, and *nad6*) use the canonical termination codons (TAA or TAG), while *nad3* and *nad5* stop with the incomplete termination signal T. The length of 22 tRNAs ranged from 52 bp (*trnA*) to 69 bp (*trnI*), A + T content ranged from 62.26% (*trnR*) to 83.64% (*trnG*). Thirteen tRNAs lacked the potential to form the cloverleaf-shaped secondary structure. Ten of them (*trnD*, *trnF*, *trnK*, *trnR*, *trnT*, *trnW*, *trnE*, *trnG*, *trnL_1_*, and *trnM*) lacked the TΨC arm stems, three tRNAs (*trnA*, *trnS_1_* and *trnS_2_*) lost the dihydrouracil (DHU) arm. The *rrnL* was located between *trnL_1_* and *trnV*, while the *rrnS* resided between *trnV* and *trnQ*. The lengths of *rrnL* and *rrnS* were 1,018 bp and 694 bp, and their A + T contents were 78.78% and 76.95%, respectively. The control region was located between *trnQ* and *trnM* with 492 bp long and 79.47% A + T content.

To validate the evolutionary position of *A. ocula*, a neighbor-joining phylogenetic tree inferred from mitochondrial 13 PCGs of 15 spiders was constructed by MEGA 7.0 (Kumar et al. 2016). The result showed that *A. ocula* had a sister relationship with *A. perforata* (Figure 1), which was in accordance with conventional morphological classification.

**Figure 1.**
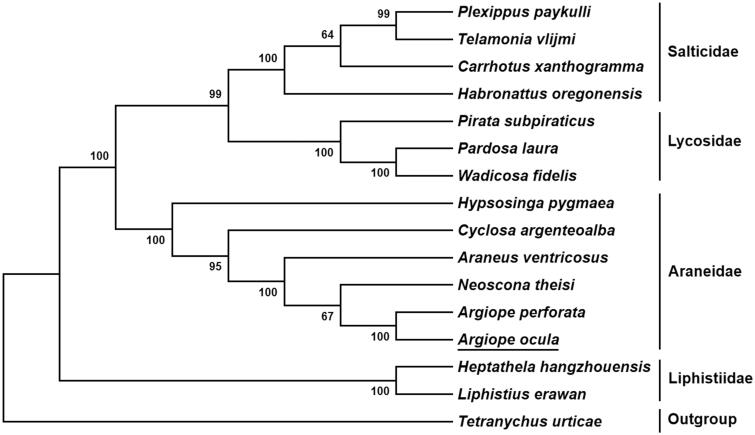
Phylogenetic tree showing the relationship between *Argiope ocula* and 14 other spiders based on neighbor-joining method. GenBank accession numbers used in the study are the following: *Araneus ventricosus* (KM588668), *Argiope perforata* (MK512574), *Carrhotus xanthogramma* (NC_027492.1), *Cyclosa argenteoalba* (KP862583), *Habronattus oregonensis* (AY571145), *Heptathela hangzhouensis* (AY309258), *Hypsosinga pygmaea* (KR259803), *Liphistius erawan* (NC_020323.1), *Neoscona theisi* (NC_026290.1), *Pardosa laura* (NC_025223.1), *Pirata subpiraticus* (NC_025523.1), *Plexippus paykulli* (NC_024877.1), *Telamonia vlijmi* (NC_024287.1), *Tetrancychus urticae* (EU345430.1), and *Wadicosa fidelis* (NC_026123.1). *T. urticae* was used as an outgroup. Spider determined in this study was underlined.

## References

[CIT0001] Kumar S, Stecher G, Tamura K 2016 MEGA7: molecular evolutionary genetics analysis version 7.0 for bigger datasets. Mol Biol Evol. 33:1870–1874. 27004904 10.1093/molbev/msw054 PMC8210823

[CIT0002] Levi HW 2004 Comments and new records for the American genera *Gea* and *Argiope* with the description of a new species (Araneae: Araneidae). Bull Mus Comp Zool. 158:47–65.

[CIT0003] Li C, Wang ZL, Fang WY, Yu XP 2016 The complete mitochondrial genome of the orb-weaver spider *Cyclosa argenteoalba* Boes. et Str. (Araneae: Araneidae). Mitochondrial DNA A DNA Mapp Seq Anal. 27:2537–2538. 26017043 10.3109/19401736.2015.1038793

[CIT0004] Platnick NI 2015 The world spider catalog, version 15.0. American museum of natural history. http://research.amnh.org/iz/spiders/catalog/.

[CIT0005] Yang WJ, Xu KK, Yang DX, Li C 2019 Characterization of complete mitochondrial genome of *Evarcha coreana* (Araneae: Salticidae). Mitochondrial DNA Part B. 4:1321–1322.

